# Speech recognition datasets for low-resource Congolese languages

**DOI:** 10.1016/j.dib.2023.109796

**Published:** 2023-11-10

**Authors:** Ussen Kimanuka, Ciira wa Maina, Osman Büyük

**Affiliations:** a Department of Electrical Engineering, Pan African University Institute for Basic Sciences, Technology and Innovation, Nairobi, Kenya; b Department of Electrical Engineering, Dedan Kimathi University of Technology, Nyeri, Kenya; c Centre for Data Science and Artificial Intelligence (DSAIL), Nyeri, Kenya; d Department of Electrical and Electronics Engineering, Izmir Demokrasi University, Izmir, Turkey

**Keywords:** Automatic speech recognition, Pre-trained models, Transfer learning, Self-supervised learning, Cross-lingual acoustic model, Multilingual acoustic model

## Abstract

Large pre-trained Automatic Speech Recognition (ASR) models have shown improved performance in low-resource languages due to the increased availability of benchmark corpora and the advantages of transfer learning. However, only a limited number of languages possess ample resources to fully leverage transfer learning. In such contexts, benchmark corpora become crucial for advancing methods. In this article, we introduce two new benchmark corpora designed for low-resource languages spoken in the Democratic Republic of the Congo: the Lingala Read Speech Corpus, with 4 h of labelled audio, and the Congolese Speech Radio Corpus, which offers 741 h of unlabelled audio spanning four significant low-resource languages of the region. During data collection, Lingala Read Speech recordings of thirty-two distinct adult speakers, each with a unique context under various settings with different accents, were recorded. Concurrently, Congolese Speech Radio raw data were taken from the archive of broadcast station, followed by a designed curation process. During data preparation, numerous strategies have been utilised for pre-processing the data. The datasets, which have been made freely accessible to all researchers, serve as a valuable resource for not only investigating and developing monolingual methods and approaches that employ linguistically distant languages but also multilingual approaches with linguistically similar languages. Using techniques such as supervised learning and self-supervised learning, they are able to develop inaugural benchmarking of speech recognition systems for Lingala and mark the first instance of a multilingual model tailored for four Congolese languages spoken by an aggregated population of 95 million. Moreover, two models were applied to this dataset. The first is supervised learning modelling and the second is for self-supervised pre-training.

Specifications Table

**Table:** 

Subject	Deep Learning, Self-Supervised Learning, Natural Language Processing, Speech processing
Specific subject area	Automatic Speech Recognition for Low-Resource languages
Type of data	Speech and audio, text
How the data were acquired	The process of collecting speech and audio for this dataset involved two sets of data: (1) Instructing participants to sit in a relaxed manner within centimetres of an audio recording device or smartphone and read from the text utterances. To capture the speech utterances, a smartphone equipped with a mobile App was utilised. This enabled precise and controlled speech capture, ensuring the dataset would contain high-quality audio that accurately depicts the uttered text; (2) Recording from the archives of a broadcast station. News and Radio programmes are aired in many Niger-Congo B languages, including Lingala, Tshiluba, Kikongo and Congolese Swahili. Crawling the websites of the Radio archives (RA) to get the URLs of audio recordings in 4 Congolese languages simplified the gathering procedure. We not only curated material from the radio's official website but also from YouTube clips that the stations had released under a Creative Commons license.
Data format	All recordings utilise Waveform Audio File Format (WAVE), and each file is encoded with a sample rate of 16 KHz on a single track (Mono).
Description of data collection	We collected and pre-processed two datasets involving 4 Congolese languages: the 4.3 h Lingala Read Speech Corpus, which can be used to create a supervised speech recognition module, and the 741 h Congolese Radio Corpus, which is designed for unsupervised speech representation learning for downstream tasks.
Data source location	Democratic Republic of the Congo For Lingala Read Speech Corpus: Quota sampling covering all locations in the Kinshasa area. For Congolese Speech Radio Corpus: Convenience sampling of online radio archives with the permission of the broadcasting station. Kinshasa
Data accessibility	The datasets can be accessed at https://data.mendeley.com/datasets/28×8tc9n9k/1 and it is freely available to the public for research, academic or educational purposes. **DOI**:10.17632/28×8tc9n9k.1

## 1 Value of the Data

• The availability of this data will assist researchers in developing more innovative techniques to enhance the low-resource Congolese Automatic Speech Recognition (ASR) system.
• Advancement in this field greatly benefits society as it provides the first step as part of many conversational Artificial Intelligence (AI) systems (i.e., Human-Computer Interaction, Natural Language Understanding NLU and Virtual assistant). In these conversational AI, ASR first translates speech into text, and then the transcription is sent to the NLU, generating responses. The responses can then be used for other applications, such as conversion to speech using text-to-speech synthesis. Unfortunately, Congolese languages tend to fall into the “low-resource” category, which, in contrast to “high-resource” languages, has fewer datasets accessible, hence limiting the development of Conversational AI.
• The Lingala Read Speech Corpus and the Congolese Radio Corpus datasets serve as a foundation for the research community, providing a starting point for further exploration and development. It serves as a valuable resource upon which researchers could build because the datasets can be enhanced by incorporating additional speech and audio.
• These two datasets open up a plethora of new avenues for future study and development. It offers potential advantages in both supervised and self-supervised speech processing and artificial intelligence applications.
• The datasets consist of supervised speech and unsupervised raw audio captured in real-world conditions, reflecting the challenges faced in practical scenarios. This realism helps in training models that can perform well in real-life situations where ambient conditions and background noises may vary.
• This will benefit speakers of 4 Congolese languages (Lingala, Tshiluba, Kikongo and Congolese Swahili) as well as those developing speech-processing software to serve as a communication tool for the under-represented group.

## 2 Objective

For the development of high-quality ASR systems, many contemporary ASR models depend on vast quantities of labelled data for each language. Such techniques are costly and not scalable, restricting the use of ASR technology to a subset of languages and populations. In addition to these issues of the availability of labelled data, Congolese languages confront a number of other issues that must be addressed. The extensive tones and diacratics found in sub-Saharan African languages make it more difficult to represent phonotactic structures; most of these languages have less digital content on the web, which poses obstacles to adopting Natural language processing (NLP) models. Likewise, there are prospects from a united standpoint as numerous sources of representation may be shared across different languages from the same family, and the collection of unlabelled data for pretraining may be used to construct multilingual models in which transfer learning is successful.

The main objective of the datasets is to facilitate increased accessibility for supervised labelled and self-supervised unlabelled corpus towards simplifying benchmarking and creation of baselines for Congolese languages as well as creation of multilingual acoustic model combining four Congolese languages (i.e., Kikongo, Tshiluba, Lingala and Congolese Swahili) The datasets aim to empower researchers and developers to create automated systems that can accurately recognize and understand the low-resource Congolese languages. This type of automated system can transcribe speech into text, making it easier for the development of conversational AI for Congolese languages without making any errors. The datasets can also be used for training expert systems, opening up new avenues for study and development and offering advantages in machine learning and artificial intelligence applications. Overall, the datasets hold promise for initial development work on benchmarking an ASR system for Congolese languages and building a multilingual acoustic model for Congolese languages, as well as advancing technological applications in the field of speech processing for low-resource Congolese languages.

## 3 Data Description

**Fig. 1:**
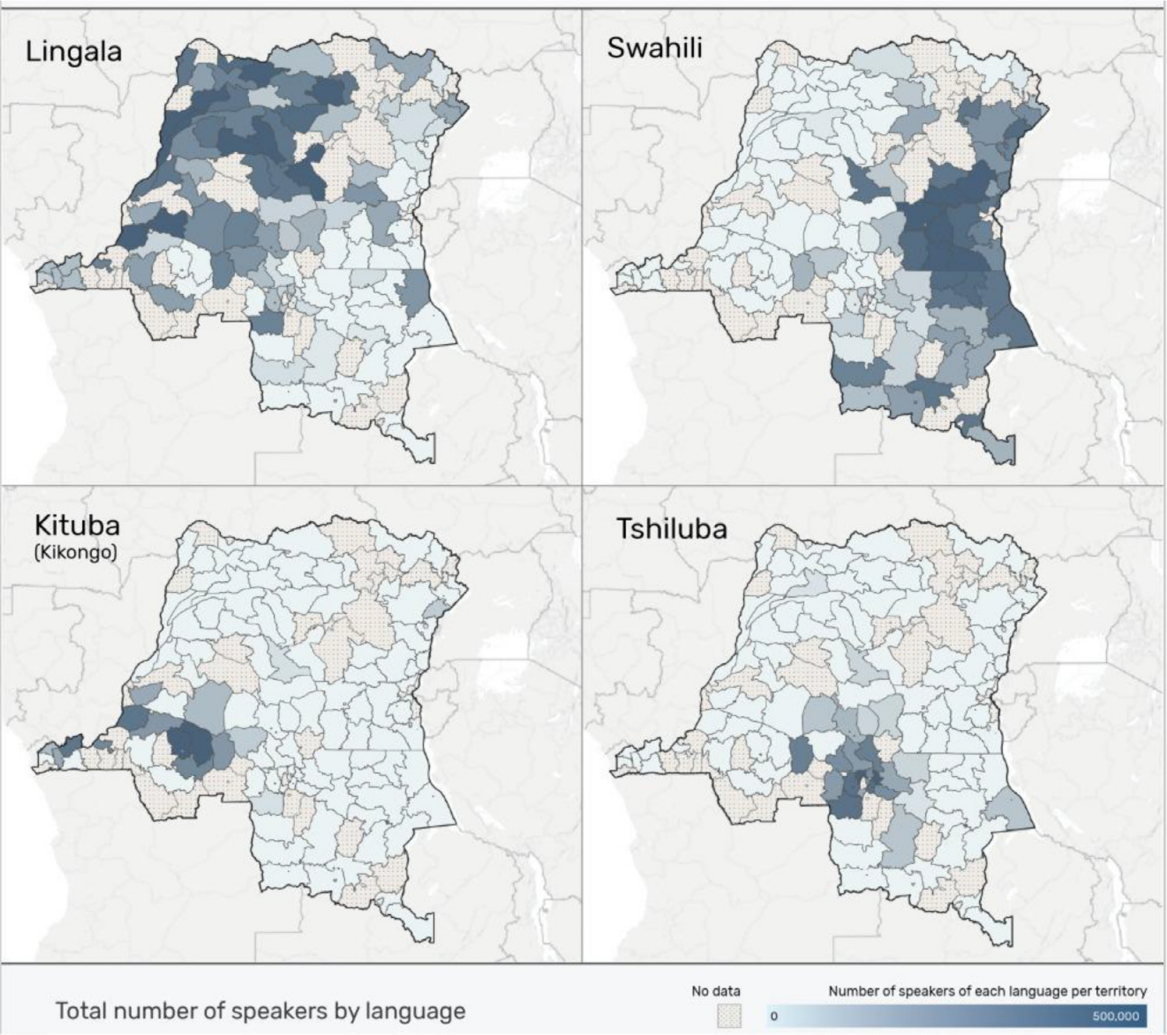
This map shows the distribution of speakers of each language in each of the country's territories in 2020 (Translators without Borders), adapted from https://bit.ly/3fYVAoc.

There are more than 200 languages spoken in the Democratic Republic of the Congo^1^; amongst them, four are constitutionally recognized since they are the top four languages in terms of global use. These are Lingala, Kongo or Kikongo, Luba-Kasai or Tshiluba, and Congolese Swahili. All belong to one language family—the Niger-Congo B family. As it is known that languages within a language family have large linguistic similarities in their acoustic phonetics, articulatory phonetics, grammatical structures, and vocabulary patterns, they also share similar characteristics such as the utilization of prefixes, suffixes, and infixes to show grammatical connections; have a sophisticated system of noun classes and verb conjugation; and also have a tonal system, agglutinative morphology, subject verb object word order, complex syllable structure, and vowel harmony. Three of the four languages in this study are written with the standard 26 letters of the Latin alphabet, except for Lingala, which uses two additional special characters: the open vowels ε and ɔ. In three of the four languages, diacritical marks can be used to indicate certain linguistic features, such as time, vowel length, or emphasis. A summary of the number of speakers, some phonological features, and orthographic conventions used for the four languages are given in Table 1 and Fig. 1 shows the reach of different Congolese languages.

**Table 1:** Numbers of speakers, phonological features and orthographic conventions.

Languages	ISO	Population	Tones	Diacratical	Example
Lingala	ln	40M	high, low	Yes	lεlɔ 'today'
Kongo/Kikongo	kon	6.9M	high, low	Yes	mbó 'to hit'
Congolese Swahili	swc	11M	high, low	No	mtoto ‘child’
Luba-Kasai/Tshiluba	lua	7M	high, mid, low	Yes	kutúla 'finished'

This section presents the process of collecting and pre-processing two datasets involving 4 Congolese languages: the Lingala Read Speech Corpus, which can be used to create the supervised speech recognition module, and the Congolese Radio Corpus, which is designed for unsupervised speech representation learning for downstream tasks.

### 4.1 Lingala Read Speech Corpus

#### 4.1.1 Description

The corpus contains a total of 4.3 h of voice data. In order to allow future experimentation, we offer consistent training, development, and testing divides. There is no speaker overlap between the subgroups. All recordings utilise Waveform Audio File Format (WAVE), and each file is encoded with a sample rate of 16 kHz on a single track (mono).

#### 4.1.2 Collection of the dataset

Lingala has a minimal online presence and few printed books; thus, we have compiled and merged a modest collection of works, including fiction, poetry and proverbs, from online and open-source hardcopy books. We performed text standardization following a more intricate and comprehensive set of rules than those defined on ASCII characters, such as fixing spelling and grammar problems, expanding abbreviations, deleting foreign terms, textually transcribing numerals and separating concatenated words. By applying these rules, we normalize the Lingala texts in different styles into a standardized form, which is a best-effort attempt to penalize only errors in transcription, not those caused by differences in formatting or punctuation. As a text corpus, we acquired 22,080 phrases, which are utilised to create lexical dictionaries and train language models. To create the Lingala Read Speech corpus, approximately 4500 phonetically balanced phrases from the acquired text corpus are selected and recorded. We the Lig-Aikuma application^2^ for speech recording. The software's elicitation mode was used to capture their reading of text scripts tokenised at the sentence level. Numerous scholars have utilised the Lig-Aikuma in their similar works [1,2]. Compared to other speech corpora, which include tens or more hours of labelled speech data for training, this corpus is relatively small, representing a low-resource scenario. As a result, the modelling will be hampered by a lack of training data. Table 2 depicts the distribution of training, development and test sets.

**Table 2:** General characteristics of the Lingala Read Speech corpus.

Subset	Duration (hours)	Utterances
Train	4	2465
Dev	0.2	204
Test	0.1	180
Total	**4.3**	**2849**

#### 4.1.3 Speakers

Lingala Read Speech recording speakers were from the Kinshasa area. There are 2849 recordings recorded by 32 distinct speakers (13 male and 19 female). The ability to speak and read Lingala was a requirement for all speakers. Noting that the recordings in this corpus were not made under perfect conditions, the speakers' voices were recorded in various settings and with different accents. Consequently, it is anticipated that some utterances will include some ambient noise. This enables us to train and evaluate ASR systems in environments that more closely mimic the real world than a studio setting.

#### 4.1.4 Pre-processing

**Fig. 2:**
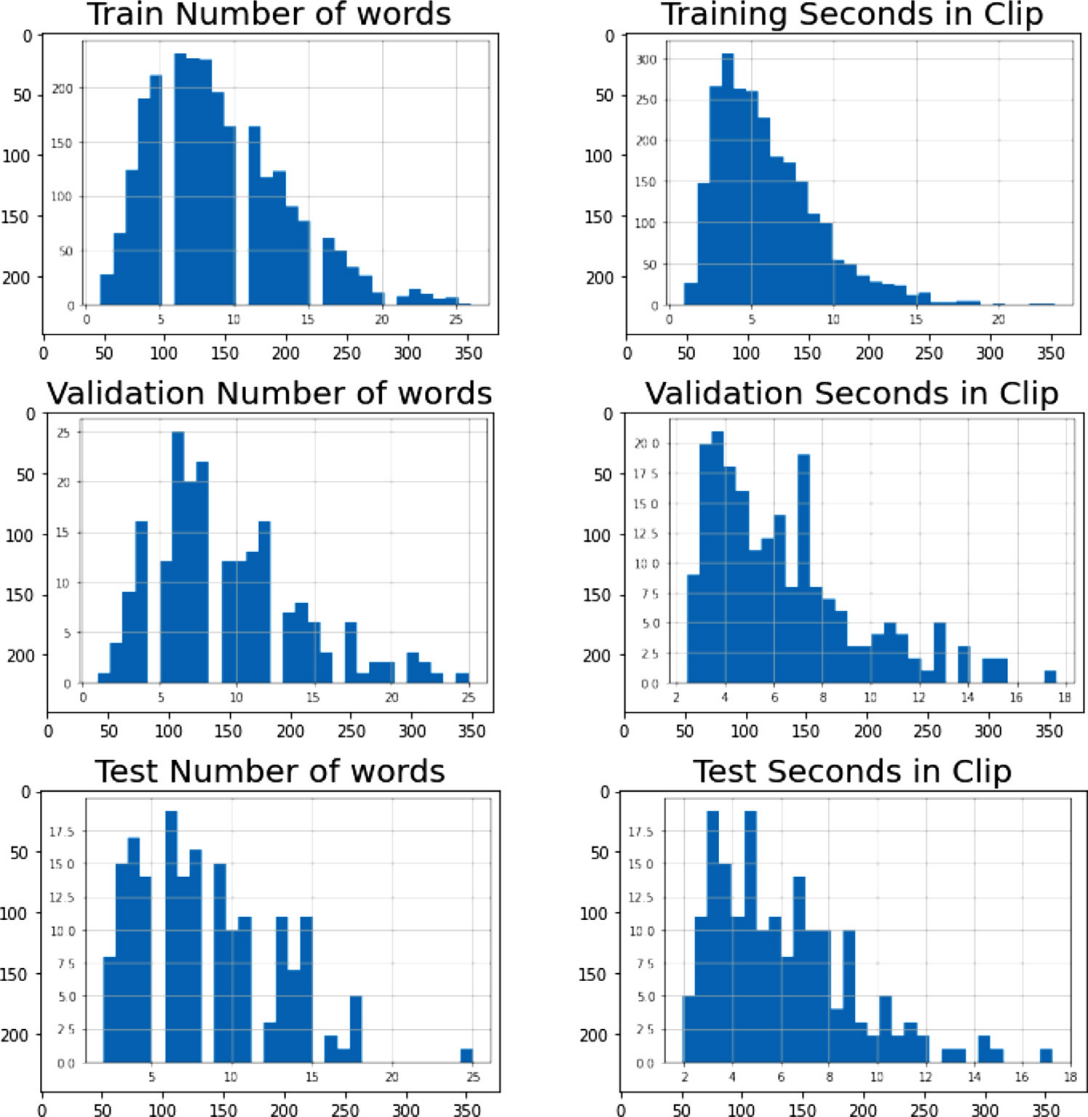
Distribution of the Lingala Read Speech Corpus dataset.

All utterances were validated against the transcripts to ensure the accuracy of the data. We also used speaker ID to sort the recorded audio files into groups based on the speaker. We also renamed all the audio files by appending the speaker ID to the utterance IDs. Fig. 2 shows the distribution of the dataset.

#### 4.1.5 Text sources

The recorded phrases and sentences are drawn from various Lingala language sources, including literature. Table 3 summarises the textual contributions to the Lingala Read Speech corpus and Table 4 the distribution of text used for language modelling tasks. Each phrase ranges in length from one to twenty words.

**Table 3:** Sources of text contained in the Lingala Read Speech corpus. The Lingala literature includes publicly available books, magazines and training materials written in Lingala. Other online resources include various websites with Lingala content.

ID	Source	Size
1	Lingala literature	80 %
2	Other online resources	20 %

**Table 4:** The token counts for the two sets of text sources used to create the language models task. LMsmall relates to texts originating from the transcript of the corpus and LMlarge are texts from additional online resources.

LM	Sentences	Tokens
LM_small_	4	2465
LM_large_	0.2	204

#### 4.1.6 Availability

The corpus will be accessed at our data repository and shared with the research community under a Creative Commons license.

### 4.2 Congolese speech radio corpus

#### 4.2.1 Description

There are 741 h of audio clips in the Congolese Radio Corpus, with the longest being 25 s long. These recordings were taken from the archives of a broadcast station in the Democratic Republic of the Congo.^3^ News and Radio programmes are aired in many Niger-Congo B languages, including Lingala, Tshiluba, Kikongo, and Congolese Swahili. There may be a variety of sounds and music playing in the background and the front of particular radio content. Crawling the websites of the Radio archives (RA) to get the URLs of audio recordings in 4 Congolese languages simplified the gathering procedure. We not only curated material from the radio's official website but also from YouTube clips that the stations had released under a Creative Commons License. Although this drastically increased the quantity of data we could gather, particularly in the low-resource Niger-Congo language family, it was necessary to guarantee that we could freely share the URLs with the scientific world, assuring the repeatability of our study.

#### 4.2.2 Pre-processing

**Fig. 3:**
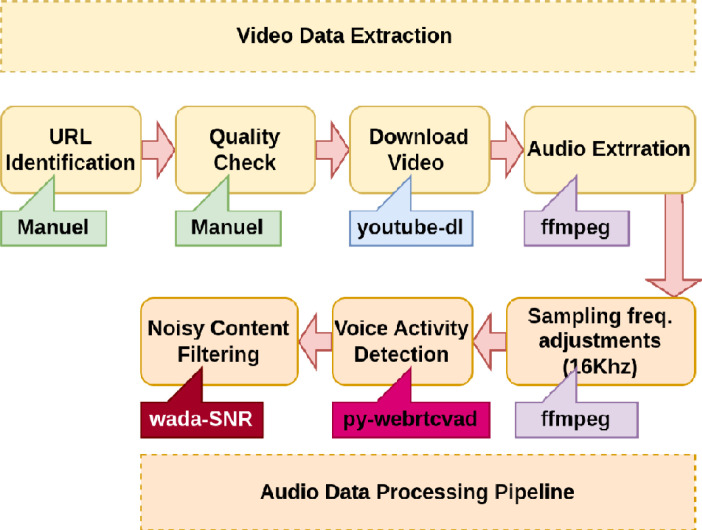
Summary of the pre-processing steps for unlabelled data.

To begin, we grabbed the audio clips from the websites of the radio broadcast; for those files hosted on YouTube, we utilised the Youtube-dl library.^4^ Secondly, the information was not always mono channel, and the sampling frequency ranged from 8 kilohertz to 44 kilohertz since the data was picked from a wide variety of sources. We used the FFmpeg library^5^ to upsample/downsample the data that was captured at a frequency of 16 kHz and then decrease the number of audio channels to 1. Thirdly, we further improved the data by excluding extended periods of silence from the audio files using the Py-webrtcvad library,^6^ a python interface to the widely used WebRTC VAD (Voice Activity Detection) module built by Google. The VAD algorithm screens out noise and lets us choose a harshness parameter (a number between 0 and 3), which determines how strictly to apply the filter on speech (0 is the least aggressive about filtering out non-speech, 3 is the most aggressive). Similar to the findings in [3], we found that changing this value to 2 provided the best results for our data. Also, we utilised Waveform Amplitude Distribution Analysis signal-to-noise ratio (SNR) to exclude audio samples with an SNR below 15 dB from our dataset. Using a sample of the audio files, a threshold was found to be optimal. Finally, we followed industry standards by chunking audio recordings to a maximum of 25 s in length. Fig. 3 summarises the pre-processing steps, and Table 5 provides a summary of the data acquired in this way.

**Table 5:** The number of hours of Congolese Speech Radio Corpus per language post processing.

Languages	Radio Archives (hours)	YouTube (hours)	Total (hours)
Lingala	187.6	86.4	274.1
Kikongo	174.4	–	174.4
Tshiluba	112.8	–	112.8
Congolese Swahili	191.1	**–**	191.1

#### 4.2.3 Availability

In a written agreement form with the broadcasting stations attached as supplementary materials to this paper, we ensured that the URLs of audio recordings would be freely shared. The audio file URLs and the scripts used to collect the data and clean it up can be accessed in the data repository.

## 5 Experimental Design, Materials and Methods

The proposed system was made up of two phases:

(a) Refining raw audio
(b) Data analysis

## 6 Refining Raw Audio

This dataset has two sections for creating two distinct types of models: the Lingala Read Speech Corpus for creating a supervised speech recognition module and the Congolese Radio Corpus, which is designed for self-supervised pretraining. The first dataset is labelled with each speech having its corresponding transcription, while the second dataset is a collection of pre-processed raw audio data. The Congolese Speech Radio corpus dataset was categorized into one main category named ‘RAW_DATA’ and a sub-category named ‘DATA_REFINED’. The ‘RAW_DATA’ directory contains unprocessed audio retrieved from the radio broadcast archives. These raw audios are of length varying between 6 and 20 min, and their total size is 200GBs before compression. Due to storage constraints, the audio file URLs, as well as the scripts used to collect the raw data and clean it up, are made publicly available under this directory. In the ‘DATA_REFINED’ folder, the raw audios were processed for voice activity detection and noisy content filtering, resulting in chunked versions of audio clips, with the longest being 25 s long.

## 7 Data Analysis

Deep learning models were employed in both parts to evaluate model development potential with this dataset. In addition to presenting and analysing the fundamental implications of these novel data corpus, our ASR experiments have the goal of achieving the following three primary goals: Firstly, these experimental analyses serve as benchmarks for new studies. Secondly, we show to which extent an unlabelled resource complements the labelled set. Additionally, we provide the performance of self-supervised multilingual and cross-lingual methods and older modelling techniques.

We begin our experiments by constructing acoustic models based on the Gaussian mixture model (GMM). GMMs are no longer the focus of ongoing studies due to their requirements of heavily engineered processing stages of hybrid combinations. Nonetheless, they are often required in hidden Markov model (HMM) dependant speech recognition. To demonstrate the efficacy of the supervised data in optimising GMM, we developed a multi-stage GMM recipe for the supervised dataset. We further implement a deep neural network (DNN) audio model as well as an attention-based encoder-decoder (AED) system for the same supervised dataset. Last but not least, we used the state-of-the-art Wav2Vec 2.0 model, which produced benchmark results for massive pre-trained models on the unlabelled dataset. All the experiments' recipes are hosted on GitHub.^7^ All the experiments are trained on Nvidia Tesla v100 16GB.

### 7.1 Baselines

We benchmarked our supervised corpora using the following baseline models in order to verify their validity:

• **HMM-GMM:** Using the supervised Lingala Read Speech Corpus training set, we began our model construction efforts with the traditional HMM-GMM optimisation. The development set of this supervised corpus was utilised for tuning the model's hyperparameters, while the test set was utilised to assess the trained model.
• **TDNN:** To investigate how model architecture affects the performance of this supervised corpus, we trained a smaller TDNN model on the Lingala Read Speech training set. Table 6 demonstrates the potential of utilising this small dataset for further model construction.

**Table 6:** Different Baseline Models and a proposed Pre-trained Model on the Lingala Read Speech Corpus LRSC and The Congolese Speech Radio corpus datasets.

Acoustic Model	Pre-trained	CER(%)	LRSC WER(%)
*Traditional ASR* Monophone GMM	-	49.3	23.3
Delta+delta-delta GMM	–	44.4	20.5
LDA+MLLT GMM	–	44	20.3
LDA+MLLT+SAT GMM	–	39.9	15.9
T-DNN	–	33.2	13.3
*End-to-End(sequence-to-sequence) ASR* AED-LAS (CRDNN + GRU)	-	99.5	100
AED-Transformers (s2t transformer small)	–	98.42	100
AED-Transformers (s2t transformer small)	Init. EN	75.24	-
*Preexisting Pre-trained Models* XLSR-53	Yes	24	6.8
XLS-R-0.3B	Yes	25.8	7.0
MMS-1B-ALL	Yes	**16.5**	-
*Proposed Pre-trained Models* CdWav2Vec (mono)	cd_mono_	22.1	6.8
CdWav2Vec (multi)	cd_multi_	**21.4**	6.81

*Note:* init. EN implies cross-linguality with English. cdmono implies monolinguality with one Language (Lingala in this case) and cdmulti implies multi-liguality with 4 Congolese languages.
• **AED:** The comprehensive assessment of the HMM-GMM and TDNN systems provides a solid foundation on which to evaluate end-to-end models (AED). The AED experiments are consistent with its current trends of being labelled data greedy [4].
• **Cross-lingual:** We utilised the concept of cross-lingual pre-training of AEDs from [5] and re-implemented it to our Lingala read speech corpus. Accordingly, the main-training model was initialised with pre-trained model weights obtained from the source language data (external resource). This cross-lingual setup allows some improvement in the AED baseline.

### 7.2 Self-supervised model

Using the unlabelled audio data given in the Congolese Speech Radio Corpus, we implemented the pretraining and fine-tuning of the self-supervised ASR model for Congolese languages:

• **Pretraining a Wav2Vec 2.0 model from scratch (CdWav2Vec)** We pre-train only on the BASE model. This BASE model is made up of 12 Transformer blocks, 768 model dimensions, 3072 FFN dimensions, and 8 attention heads. In the quantization module of this design, we use *G* = 2(codebooks) with *V* = 320 elements per codebook. We have used the pre-trained checkpoint of the equivalent (BASE) English Wav2Vec model to kick off our pretraining process. We next use our preprocessed Congolese Speech Radio archive dataset to further pre-train the model. Shorter audio clips of 15.6 s in length (256k samples) are used in the BASE model.
• **Fine-tuning a Wav2Vec 2.0 model** During fine-tuning, we update all of the network parameters besides the convolutional features encoder parameters. In our fine-tuning, we considered three scenarios: (1) Fine-tuning the CdWav2Vec model pre-trained on one amongst the 4 Congolese languages (Monolingual or Cross-lingual setup) (2) Fine-tuning the CdWav2Vec model pre-trained on the 4 Congolese languages (Multilingual setup); (3) Fine-tuning preexisting multilingual XLSR-53, XLS-R and MMS models. We explored experiments on the XLS-R-0.3B model with 600 million parameters due to resource constraints circumstances. Both the fine-tuning used the supervised Lingala Read Speech Corpus implemented using either the HuggingFace transformer library [6] or the Fairseq toolkit [7]. The hyperparameters used for pretraining and fine-tuning are similar to the BASE model in [8]. As shown in Table 6, the three Wav2Vec 2.0 models (XLSR-53, XLS-R-0.3B and MMS-1B-all) and our CdWav2Vec model performance are highlighted. The latter outperforms the XLSR-53 and XLS-R-0.3B when fine-tuning on the Lingala Read Speech Corpus, even though these large models strictly have seen a superset of data. However, the CdWav2Vec couldn't outperform the MMS-1B-ALL [9] because this model has significantly seen 1403 languages during its pretraining.

Here our objective is to show the effectiveness of our radio archive unlabelled data.

Afterwards, we honed in on the CdWav2Vec model and assessed it on 3 test sets from 2 Congolese languages and 3 language model configurations. According to some research, LM is done because transcripts make a decent language model, but adding available in-domain text data is even better. Hence, Table 7 shows the in-domain and out-of-domain outcomes of our trained models. One test set is in-domain (test subset of the Lingala Read speech corpus) while the other two are out-of-domain: (1) The TICO-19 test set consists of read speech of Congolese Swahili recorded from the dev set of the machine translation benchmark for COVID-19 domain [10] and (2) the Fleurs Lingala test set, provided as part of Google research as the speech version of the Flores machine translation benchmark [11] for the evaluation ASR models. The 3 LM configurations are the LM_small_ (only the train subset of the transcription are used for LM training), LM_large_ (crawled text from news websites are used for LM training) and LM_general_ (general text from different topics are crawled from different sources for LM training). The results for the test sets show how different the in-domain and out-of-domain LM influences the WER. From Table 7, we can see that the domains of the Lingala Read Speech and Fleurs test sets are much closer to the pretraining and finetuning corpus. Hence CdWav2Vec AM performs identically for the in-domain test and out-of-domains. Finally, it can be observed that regardless of being pre-trained on 100 times less unlabelled data, the CdWav2Vec model using Congolese Speech Radio Corpus performs on par with the highly massive MMS-1B-ALL on the out-of-domain fleurs Lingala subset test set.

**Table 7:** In-domain and out-of-domain analysis. The unlabelled Radio Corpus was utilized in one model. Scores are presented in WER and CER format after being categorized by the test set.

AM	Transcript	LM_small_	LM_large_	LM_general_
*LRSC test set (in-domain)* XLSR-53	24	20.2	23.7	24
XLS-R-0.3B	25.8	21.9	24.7	23.9
CdWav2Vec	**21.4**	**19.1**	**18.4**	**21**
*TICO-19 SWC test set (out-of-domain)* End-to-End [12]	18.3			
CdWav2Vec	13.7	14.7	14.7	14.7
*Fleurs Lingala test set (out-of-d)* XLSR-53	25	19.9	18.9	19.3
XLS-R-0.3B	26.7	22.2	21.2	21.1
MMS-1B-ALL	-/**4.3**	-/-	-/-	-/-
CdWav2Vec	23.2/9.1	20.1/10.4	19.9/9.7	20.3/9.8

### 7.3 Monolingual vs multilingual

Using checkpoints from monolingual and multilingual pretraining, with monolingual meaning only one language from the unlabelled set used for self-supervised pretraining, we fine-tuned 4.3 h of Lingala data. Table 8 demonstrates that Lingala benefits from multilingual pretraining even with a small quantity of supervised data.

**Table 8:** Effect of multilingual and monolingual pretraining from the Congolese Speech Radio corpus.

Model	Pretraining	Finetuning	Decoding	WER	CER
CdWav2Vec(cd_mono_)	monolingual ln	Lingala	Viterbi	22.1	6.8
CdWav2Vec(cd_mono_)	monolingual swc	Lingala	Viterbi	26	7.6
CdWav2Vec(cd_mono_)	monolingual kon	Lingala	Viterbi	28.1	8.9
CdWav2Vec(cd_mono_)	monolingual tshi	Lingala	Viterbi	26.8	7.9
CdWav2Vec(cd_multi_)	multilingual	Lingala	Viterbi	21.4	6.8

### 7.4 Impact of cross-lingual representations

**Fig. 4:**
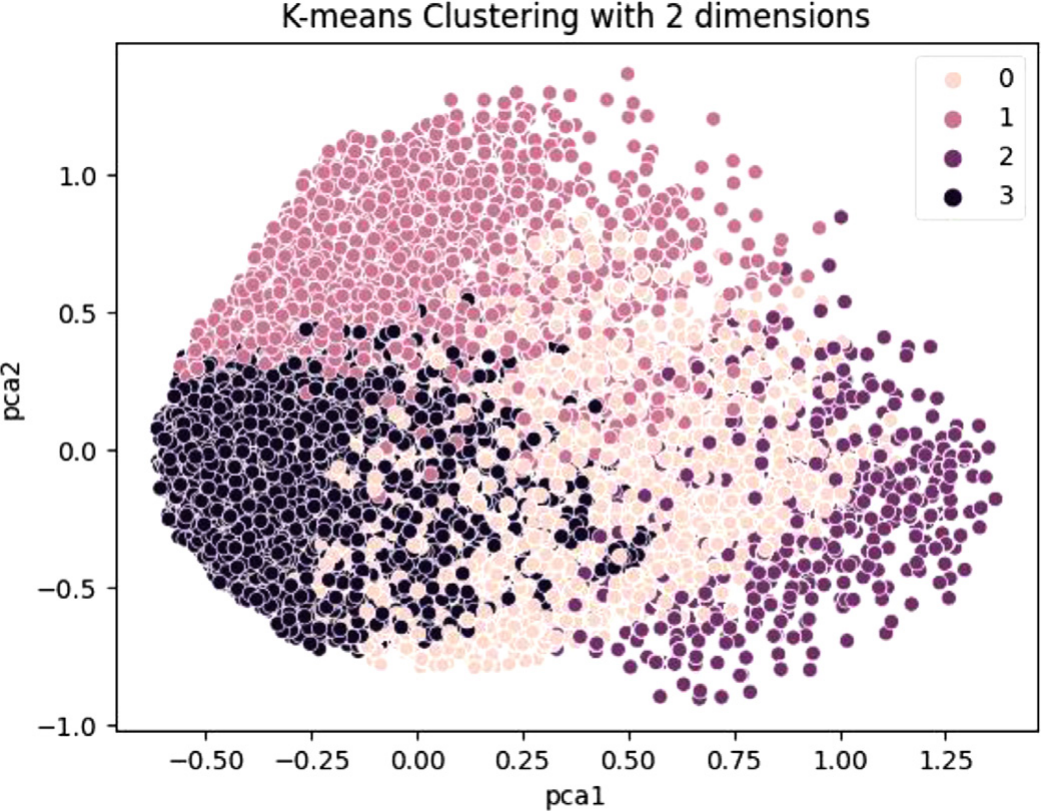
Quantized speech representation where 0: Kikongo, 1: Lingala, 2: Congolese Swahili and 3: Tshiluba.

It has been explained earlier that the first stage of the CdWav2Vec model entails calculating representations of audio frames from a learned codebook. All four languages are presented in the model using the same codebook vectors. In the same vein as [13], we seek to determine whether or not these codebooks are independent or shared across languages and whether or not related languages share codebook entries, which increase with linguistic distance. To this end, we constructed a graphic that displays the quantised speech representations for all the languages in our study. To accomplish this, 200 samples are chosen at random from each language for a grand total of 5 h of data. For the purpose of creating codebook vectors, we first run the audio through the feature encoder and then through the quantiser. For each sequence, the quantiser module's output vectors. For each language, vectors are normalised to form VXG vectors of size, followed by k-means clustering and principal component analysis to reduce the dimensions of these vectors. As shown in Fig. 4, groupings of languages that are most phonetically similar to one another tend to cluster together. This gives more evidence that multilingual pretraining with self-supervised approaches may promote representation learning that is transferable across linguistically related low-resource languages.

## Ethics Statement

The author declares that all contributors of the Lingala Read Speech Corpus voluntarily participated in its creation. No personal information, such as phone numbers or email addresses, was requested. Informed consent was obtained from all the individual participants included in the data collection process. Before recording the speech, the participants were briefed on the objectives of the data collection. In the Congolese Speech Radio Corpus, an authorization consent from the broadcasting station was acquired. No personal information that would allow identifying individuals was collected. The IRB/local ethics was not required in this setting because the Ordinance Law No. 23/008 of March 10, 2023, authorizing the ractification by the DRC of the African Union (AU) convention on Cybersecurity and the protection of personal data, and Ordinance Law No. 23/010 0f March 13 on the Digital Code in the DRC (the Digital Code), allow collection of data with the consents of the participant for academic purposes.

## CRediT authorship contribution statement

**Ussen Kimanuka:** Conceptualization, Methodology, Software, Data curation, Writing – original draft, Visualization, Funding acquisition, Investigation. **Ciira wa Maina:** Validation, Supervision. **Osman Büyük:** Writing – review & editing, Supervision.

## Data Availability

Speech Recognition Datasets for Congolese Languages (Original data) (Mendeley Data)

## Footnotes

1 http://www.ethnologue.com

2 https://lig-aikuma.imag.fr/

3 https://www.radiookapi.net/journaux-tous

4 https://github.com/tpikonen/youtube-dl

5 https://ffmpeg.org/

6 https://github.com/wiseman/py-webrtcvad

7 https://github.com/ussenuk/CdWav2Vec-f
